# Molecular Signaling Mechanisms and Function of Natriuretic Peptide Receptor-A in the Pathophysiology of Cardiovascular Homeostasis

**DOI:** 10.3389/fphys.2021.693099

**Published:** 2021-08-19

**Authors:** Kailash N. Pandey

**Affiliations:** Department of Physiology, School of Medicine, Tulane University Health Sciences Center, New Orleans, LA, United States

**Keywords:** natriuretic peptides, natriuretic peptide receptors, gene-targeting, cardiovascular disorders, genetic mouse models

## Abstract

The discovery of atrial, brain, and C-type natriuretic peptides (ANP, BNP, and CNP) and their cognate receptors has greatly increased our knowledge of the control of hypertension and cardiovascular homeostasis. ANP and BNP are potent endogenous hypotensive hormones that elicit natriuretic, diuretic, vasorelaxant, antihypertrophic, antiproliferative, and antiinflammatory effects, largely directed toward the reduction of blood pressure (BP) and cardiovascular diseases (CVDs). The principal receptor involved in the regulatory actions of ANP and BNP is guanylyl cyclase/natriuretic peptide receptor-A (GC-A/NPRA), which produces the intracellular second messenger cGMP. Cellular, biochemical, molecular, genetic, and clinical studies have facilitated understanding of the functional roles of natriuretic peptides (NPs), as well as the functions of their receptors, and signaling mechanisms in CVDs. Transgenic and gene-targeting (gene-knockout and gene-duplication) strategies have produced genetically altered novel mouse models and have advanced our knowledge of the importance of NPs and their receptors at physiological and pathophysiological levels in both normal and disease states. The current review describes the past and recent research on the cellular, molecular, genetic mechanisms and functional roles of the ANP-BNP/NPRA system in the physiology and pathophysiology of cardiovascular homeostasis as well as clinical and diagnostic markers of cardiac disorders and heart failure. However, the therapeutic potentials of NPs and their receptors for the diagnosis and treatment of cardiovascular diseases, including hypertension, heart failure, and stroke have just begun to be expanded. More in-depth investigations are needed in this field to extend the therapeutic use of NPs and their receptors to treat and prevent CVDs.

## Introduction

The natriuretic peptides (NPs) family contains a group of hormones that are pivotal in the control of cardiovascular, endocrine, renal, and vascular homeostasis (Brenner et al., 1990; Drewett and Garbers, 1994; de Bold et al., 2001; McGrath et al., 2005; Pandey, 2005a; Rubattu et al., 2006; Kishimoto et al., 2011). Atrial and brain natriuretic peptides (ANP, BNP) are the members of the NPs hormone family. A third member of the NPs family, C-type, natriuretic peptide (CNP) was also isolated and identified but each NP was found to be derived from a separate gene (Rosenzweig and Seidman, 1991; LaPointe, 2005; Schulz, 2005). ANP and BNP exhibit diuretic, natriuretic, vasorelaxant, antiproliferative, antiinflammatory, and antihypertrophic effects that are directed toward the reducing and controlling of body fluid volume, blood pressure (BP) and cardiovascular diseases (CVDs) (McGrath et al., 2005; Ellmers et al., 2007; Wang T.J. et al., 2007; Pandey, 2011; Volpe et al., 2014; Cannone et al., 2019). Although the possibility of NPs role in metabolic regulation has been given limited consideration but NPs potently affect lipid and glucose metabolism, may also contribute to the pathophysiological link between metabolic and CVDs (Schlueter et al., 2014; Jordan et al., 2018). ANP has been shown to induce lipolysis and lipid oxidation and to ameliorate insulin resistance (Birkenfeld et al., 2008; Coue and Moro, 2016). All three NPs (ANP, BNP, and CNP) have highly homologous sequence structures, bind to cognate cell surface receptors, and elicit discrete biological and physiological functions (Brenner et al., 1990; Koller and Goddel, 1992; Anand-Srivastava and Trachte, 1993; Khurana and Pandey, 1993; Pandey, 2008). Each member of the family of endogenous NPs, including ANP, BNP, CNP, and urodilatin, has an integral role in BP, renal dysfunction, and CVDs (de Bold, 1985; Levin et al., 1998; Pandey, 2011; Rubattu and Volpe, 2014).

Importantly, NPs not only regulate BP and CVDs, but also maintain natural antagonistic actions to the renin-angiotensin-aldosterone system (RAAS), exert antimitogenic effect, and inhibit myocardial hypertrophy and fibrosis. Moreover, NPs play roles in endothelial cell function, cartilage growth, immunity, and mitochondrial biogenesis (Pandey, 2005a; Vollmer, 2005; Garbers et al., 2006; Ellmers et al., 2007). The demonstration of structurally related NPs indicated that the physiological control of BP and cardiovascular homeostasis is both complex and multifactorial. Studies of a combination of cellular, biochemical, molecular, genetic, and pharmacological properties of NPs and their prototype receptors have suggested the hallmark functions with physiological and pathophysiological responsiveness, including cardiovascular, endocrine, renal, neuronal, skeletal, and immunological importance in health and disease (Kuhn, 2005; Vollmer, 2005; Kishimoto et al., 2011; Misono et al., 2011; Jordan et al., 2018; Pandey, 2018).

Different NPs receptors have been classified namely; NP receptor-A (NPRA), NP receptor-B (NPRB), and NP receptor-C (NPRC). NPRA and NPRB exhibit an intrinsic intracellular guanylyl cyclase (GC) catalytic domain and are designated as GC receptor-A (GC-A/NPRA) and GC receptor-B (GC-B/NPRB) (Drewett and Garbers, 1994; Levin et al., 1998; Pandey, 2005a). Both ANP and BNP bind and activate NPRA, which produces intracellular second messenger cGMP in response to hormone binding. CNP activates NPRB and also produces cGMP. All three NPs (ANP, BNP, CNP) bind to NPRC, which lacks a GC catalytic domain and does not increase levels of intracellular cGMP (Fuller et al., 1988; Garbers, 1992; Koller and Goddel, 1992; Khurana and Pandey, 1993; Matsukawa et al., 1999). NPRA is a principal loci involved in the regulatory action of ANP and BNP (Lucas et al., 2000; Tremblay et al., 2002; Pandey, 2005a). Determining the insight into the intricacies of ANP-BNP/NPRA/cGMP signaling is of primary importance if we are to understand both the receptor biology and CVDs arising from cell- and tissue-specific abnormal hormone-receptor interplay. The binding of ANP and BNP to the extracellular domain of NPRA seem to exerts a conformational shift or change, whereby the signal is transmitted to the intracellular GC catalytic region of NPRA, which then activates generation of the second messenger cGMP in target cells and tissues (Pandey and Singh, 1990; Garbers, 1992; Koller and Goddel, 1992).

Although, great importance has been placed on the functional roles of NPs and their receptors in renal, cardiovascular, endocrine, neuronal, skeletal, and adipose homeostasis, in-depth research is still attempting to fully understand their potential molecular and therapeutic targets in the diseases states. We expect that future research on NPs and their receptors will yield new therapeutic targets and novel loci for the prevention and treatment of hypertension, stroke, and cardiovascular events. Earlier studies focused on elucidating the biochemical, cellular, molecular, and genetic aspects of the mode of functioning of NPRA, which still are not fully understood (Kishimoto et al., 2011; Misono et al., 2011; Pandey, 2011). Studies of cultured cells *in vitro* and transgenic and gene-targeted (gene-knockout and gene-duplication) mouse models *in vivo* have greatly advanced our understanding of NPs and their receptors by delineating the normal and abnormal control of physiological and pathophysiological functions in CVDs (Pandey, 2005a, 2019; Ellmers et al., 2007; Volpe et al., 2019a).

## Natriuretic Peptide Hormone Family

Almost four decades ago, it was firmly established that atrial homogenate contains diuretic and natriuretic activity and simultaneously identified and characterized the hormone atrial natriuretic factor (ANF) now designated as ANP in cardiac myocytes (de Bold et al., 1981; de Bold, 1985). ANP, the initial member of the NP hormone family, is predominantly synthesized and secreted from the cardiac atrium, which led to classification of the heart as an endocrine organ. The primary structure deduced from the cDNA sequence, demonstrated that ANP is synthesized as a 152-amino acid pre-pro-ANP that contains the sequences of biologically active hormone in the carboxyl-terminal region and has the major form of circulating ANP as a 28-amino acid peptide molecule (Maki et al., 1984). It was found that a 17-amino acid disulfide-bonded ring structure of circulating ANP is essential for its physiological functions. Indeed, disruption of the ring structure of ANP abolished its functional activity (Misono et al., 1984; de Bold, 1985; Brenner et al., 1990). BNP is synthesized as a 134-amino acid pre-pro-BNP that produces a 108-amino acid prohormone. Processing of pro-BNP yields a 75-residue amino-terminal-BNP (NT-pro-BNP) and a biologically active 32-amino acid circulating BNP molecule (Sudoh et al., 1988; Seilhamer et al., 1989). CNP is synthesized as a 103-amino acid pre-pro-CNP cleaved to a 53-amino acid peptide by the protease furin. It is subsequently processed to yield a 22-amino-acid biologically active CNP (Wu et al., 2003).

Cellular, biochemical, molecular, and immunohistological studies have suggested that three specific NPs (ANP, BNP, CNP) and their three distinct receptor subtypes (NPRA, NPRB, NPRC) have widespread cell and tissue distributions, indicating the pleotropic actions at the systemic and local levels (Pandey et al., 1986, 1988; Leitman et al., 1988; Brenner et al., 1990; Levin et al., 1998). ANP and BNP exhibit the most variability in primary sequence structure across species, while CNP is highly conserved among species. Subsequently, a 32-amino acid peptide, urodilatin (URO) was identified in urine. However, URO is not detected in the circulation. It appears to be a unique intrarenal NP with largely unexplored physiological functions (Schulz-Knappe et al., 1988; Saxenhofer et al., 1990; Feller et al., 1990; Goetz, 1991). Immunohistological staining has indicated that URO is mainly synthesized in renal cortical tubules around the collecting ducts; however, its exact role has yet to be determined (Sugimoto et al., 1993; Forssmann et al., 1994; Meyer et al., 1996). Additionally, another member of the NPs hormone family, the D-type natriuretic peptide (DNP), was initially isolated from the venom of green mamba (*Dendroaspis angusticeps*) as a 38-amino acid biologically active peptide molecule (Schweitz et al., 1992; Lisy et al., 1999). ANP shows a rapid clearance rate in the circulation; its half-life ranges from 0.5 to 3.5 min in experimental animals (Nakao et al., 1986; Yandle et al., 1986; Ruskoaho, 1992). In human, the half-life ranges between 2 and 2.5 min (Nakao et al., 1986). The clearance of BNP in humans occurs with both a short half-life of 3–4 min and a long half-life of 20–23 min (Mukoyama et al., 1991; Holmes et al., 1993). CNP has a half-life of 2−3 min in humans and approximately 1.5−2.0 min in experimental animals (Hunt et al., 1994; Charles et al., 1995).

The design of chimeric NPs has led to synthesis of the biologically active novel NPs of clinical importance, which represent single-chemical molecule with combined structural and functional properties (Lisy et al., 2008; Ichiki et al., 2019). Chimeric NPs exert the actions of more than one NP molecule, often with reduced undesirable or adverse hypotensive effects. Previously, a chimeric NP, namely CD-NP containing 22 residues of CNP and 15 residues at the carboxyl-terminus of DNP was synthesized, which showed vasorelaxant properties and cardiac and renal protective effects (Lisy et al., 2008; Lee et al., 2009; Ichiki et al., 2019). The synthetic CD-NP showed high resistance to NP degrading enzyme, neutral endopeptidases, making it clinically useful than endogenous naturally occurring NPs (Ichiki et al., 2019). Interestingly, it is expected that endogenous GC-A/NPRA activators and designer NPs will be powerful tools for preserving renal and cardiovascular functions and decreasing mortality among patients with cardiac events (Chen et al., 2019).

## Natriuretic Peptides Levels in Humans

The cardiac hormones, ANP and BNP are released from the heart in response to atrial stretch and distension. The ANP concentration ranges at the level of 50−100-fold greater than BNP. The primary sites of synthesis and secretion of ANP and BNP is cardiac atrium. Both ANP and BNP are also synthesized in the ventricle; however, 100−1,000-fold lower than does the atrium. In cardiac disease states, the ventricle becomes the primary site of synthesis and release of BNP. A recent study showed that super-enhancer cluster controls the *Nppa* and *Nppb* promoters in a competitive mode instead in a cooperative manner (Man et al., 2021). These authors suggested that the super-enhancer cluster selectively regulates the expression of *NPPA* and *NPPB* during CVDs, resulting in an increased expression of *NPPA* after the *NPPB* region is deleted, which augments the stress-induced expression of NPRA and prevention of premature cardiac hypertrophy in human. Circulating levels of ANP and BNP are greatly elevated in the early stages of cardiac infarction (Tomoda, 1988; Phillips et al., 1989; Morita et al., 1993). The expression of ANP and BNP also increases in the atrium and ventricle during the initiation of cardiac hypertrophy and congestive heart failure (CHF) (Mukoyama et al., 1991; Volpe, 2014; Nakagawa et al., 2019). In patients with severe CHF, the levels of ANP and BNP increase more than do control levels but the BNP concentration increases to a level 10−25-fold greater than fold increased in ANP levels (Mukoyama et al., 1991). A recent study found differential regulation of ANP and BNP in patients with acute decompensated heart failure (ADHF) (Reginauld et al., 2019). These authors suggested that a deficiency of ANP might exhibit a unique characteristics of CHF. However, the exact mechanism of this type of ANF deficiency with elevated BNP levels is not yet know. Those findings suggested that the heart plays a potential compromised compensatory endocrine role with differential regulation of ANP and BNP in ANP-deficient ADHF subpopulation (Reginauld et al., 2019). Previously, it had been suggested that obese hypertensive men, despite having high BP, have lower than anticipated ANP level in the plasma (Asferg et al., 2013). Similarly, a previous study indicated that a lack of compensatory ANP elevation occurs in the advanced phase of hypertension (Macheret et al., 2012). This study also showed the prevalence of impaired synthesis and secretion of BNP, pro-BNP, and NT-pro-BNP in hypertensive patents.

ANP and BNP show similar hemodynamic and physiological responses. But because of longer half-life of BNP (12−20 min) than ANP (0.5−4), BNP exhibits extended action and leads to enhanced natriuretic and diuretic actions as compared with ANP (Yoshimura et al., 1991; Omland et al., 1996). The cardiac atrium expresses almost 50–100- fold higher ANP mRNA levels than do extra cardiac tissues (Gardner et al., 1986). Although, in normal subjects; circulating BNP levels are far less than ANP levels, the increases in BNP concentrations in plasma can be 5−10-fold higher than the fold increases in the levels of ANP in CHF patients (Mukoyama et al., 1991; Hanford et al., 1994; Hanford and Glembotski, 1996). The half-life of NT-proBNP is much longer (90−120 min) than the half-life of ANP and BNP (Weber and Hamm, 2006).

Because of the longer half-life and stability, the measurement of NT-proBNP as a diagnostic marker is preferred over measurements of BNP and ANP. Moreover, the NT-proBNP molecule is considered to be a predictive marker after cardiac transplantation (Gardner et al., 2006). In contrast, CNP does not seem to behave as a cardiac hormone and its levels in the circulation are very low (Igaki et al., 1996). CNP is largely present in the vascular endothelial cells and central nervous system (Ogawa et al., 1992; Suga et al., 1992, 1993; Tamura et al., 1996; Chen and Burnett, 1998).

## Structural Domains and Signaling Mechanisms of NPs Receptors

Initially, using photoaffinity labeling and GC activity assay, three distinct types of NP receptors were identified and classified in different cell types (Pandey et al., 1988). Using molecular cloning, GC-A/NPRA was sequenced from rat brain, human placenta, and murine testis (Chinkers et al., 1989; Lowe et al., 1989; Pandey and Singh, 1990). Three distinct sub-types of NPs receptors (NPRA, NPRB, and NPRC) constitute the NPs receptor family; however, these receptors are variable in their ligand-binding specificity, GC activity, and signal transduction mechanisms (Chinkers et al., 1989; Garbers, 1992; Koller and Goddel, 1992; Khurana and Pandey, 1993). NPRC lacks the intracellular GC catalytic domain and by default, has been designated as NP clearance receptor (Maack et al., 1987; Fuller et al., 1988). Both ANP and BNP selectively activate NPRA, whereas CNP primarily binds to NPRB; all three NPs show binding affinity to NPRC (Koller et al., 1991; Suga et al., 1992; Khurana and Pandey, 1993). ANP binding to its receptor *in vivo* probably exerts a chloride-dependent feedback-control on receptor function (Misono, 2000; Misono et al., 2005). The general structural topology of NPRA and NPRB is consistent with the GC-receptor family, containing four distinct regions: an extracellular ligand-binding domain, a single transmembrane spanning region, an intracellular protein kinase-like homology domain (protein-KHD), and an intracellular carboxyl-terminal GC catalytic domain. The biologically dominant form of the NP receptors is NPRA, which is distributed in several peripheral and visceral cells and tissues and mediates most of the known physiological and pathophysiological actions of ANP and BNP.

Solubilization of the extracellular domain of NPRA provided a starting point for the studies of its ligand-binding domain (Pandey and Kanungo, 1993; Huo et al., 1999). NPRA was crystalized as a dimer of two receptor molecules with a tendency to dimerize spontaneously (van den Akker et al., 2000). Based on the crystal structure of NPRC, the hormone-induced structural changes in NPRA differ from those in NPRC. Ligand-binding to NPRC causes the dimer to bend between the two subdomains of the receptor creating a clamping motion to capture the ligand (He et al., 2001). Conformational flexibility at the intra-molecular hinge region in NPRC seems to be a critical factor for the broad-specificity of this receptor protein in binding all three NPs, including ANP, BNP, and CNP (He et al., 2001). Based on crystallographic modeling, two polypeptide chains of NPRA are required to activate the functional receptor molecule (van den Akker et al., 2000). It is predicted that the transmembrane GC receptors function as homodimers and that the dimerization region of NPRA is located between the protein-KHD and GC catalytic domains, forming an amphipathic alpha helix structure (de Lean et al., 2003; Misono et al., 2005). NPRB, which is mainly localized in the brain and vascular tissues, is thought to mediate CNP-dependent actions in the endothelial cells of the vasculature and central nervous system (Schulz, 2005). NPRC consists of a large extracellular domain of 496-amino acids, a single transmembrane domain, and a very short 37-amino-acid cytoplasmic tail that contains no homology to any other known receptor domain (Fuller et al., 1988; Matsukawa et al., 1999).

After ligand binding, both NPRA and NPRC are internalized and redistributed into subcellular compartments (Rathinavelu and Isom, 1991; Koh et al., 1992; Pandey, 1992, 2005b; Mani et al., 2015; Mani and Pandey, 2019). The endocytosis and intracellular sequestration of NPRA involves a series of sequential sorting steps through which ligand-receptor complexes are eventually internalized, sequestered, redistributed, and degraded in the lysosomes. However, a population of ligand-receptor complexes recycles back to the plasma membrane, and intact ligand is released outside the cell, while the receptor is anchored on the cell surface (Pandey, 1993; Pandey et al., 2002, 2005; Mani et al., 2015; Mani and Pandey, 2019). Trafficking of NPRB occurs in a ligand-dependent manner in response to stimulation with CNP. NPRB is internalized and recycled back to the plasma membrane (Brackmann et al., 2005). Degradation of the majority of internalized ligand-receptor complexes of NPRA, NPRB, and NPRC occurs into the lysosomes. Two specific sequence motif in the carboxyl terminal domain of NPRA, namely GDAY (Gly^920^ –Asp^921^-Ala^922^-Tyr^923^) and FQQI (Phe^790^-Gln^791^-Gln^792^-Ile^793^) have been identified, which serve as consensus internalization signal motifs for endocytosis and intracellular trafficking of this receptor protein (Pandey et al., 2005; Mani et al., 2016; Mani and Pandey, 2019). However, a specific sequence motif has not yet been identified for the internalization of either NPRB or NPRC. The internalization of NPRA also involves micro-RNA and clathrin-dependent pathways (Somanna et al., 2013, 2018).

**FIGURE 1:**
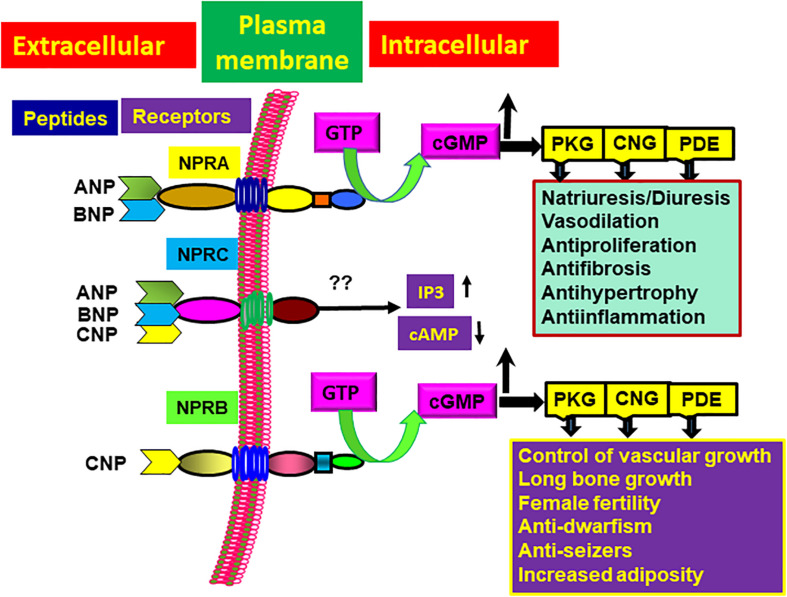
Ligand-binding specificity of different NPs (ANP, BNP, and CNP) with their cognate receptors (NPRA, NPRB, NPRC) and intracellular signaling mechanisms leading to the physiological responses. The diagram depicts that specific NP ligands activate their cognate receptor(s) and generate intracellular signals. The extracellular ligand binding domain (LBD) and intracellular protein kinase-like homology domain (protein-KHD) and guanylyl cyclase (GC) catalytic domain of GC-A/NPRA and GC-B/NPRB are shown. Both NPRA and NPRB produce intracellular second messenger cGMP, which activates effector molecules, including cGMP-dependent protein kinase (PKG), gated ion channels (CNG), and phosphodiesterase (PDE) to produce specific biological and physiological responsiveness.

Ligand-binding seems to allosterically regulate increased specific activity of the GC catalytic domains of NPRA and NPRB, which catalyzes cGMP production (Drewett and Garbers, 1994; Pandey et al., 2000; Pandey, 2005a; Burczynska et al., 2007). ANP markedly and dose-dependent increases second messenger cGMP in target cells and tissues (Waldman et al., 1984; Pandey et al., 1985, 1988, 2002, 2005). Confocal immunofluorescence analyses have demonstrated that cGMP is continuously produced in parallel during the internalization of ligand-bound NPRA in the subcellular compartments (Mani et al., 2015, 2016; Mani and Pandey, 2019). The generation of intracellular cGMP by NPRA stimulates at least three known cGMP effector protein molecules: cGMP-dependent protein kinases (PKGs), cGMP-dependent phosphodiesterases (PDEs), and cGMP-dependent ion channels (Pfeifer et al., 1998; Kaupp and Seifert, 2002; Maurice et al., 2003; Rybalkin et al., 2003; Schlossmann et al., 2005; Reinhart et al., 2006; Pandey, 2008). The activation of cGMP-dependent effector molecules leads to a wide range of signaling mechanisms leading to physiological functions, including excretion of salt and water, vasorelaxation, antiinflammation, antifibrosis, antihypertrophic actions, and immune suppressive responses (Figure 1). Overall, the signaling mechanisms of NPs and their receptors, lead to diverse physiological and pathophysiological actions, which together provide the protection to CVDs.

## NPs and Their Receptors in Cardiovascular Disorders

Several lines of evidence suggest that from the perspective of pathophysiology, a characteristic hallmark of CVDs is the elevation of ANP and BNP concentrations in patients with chest pain and cardiovascular dysfunction (Burnett et al., 1986; Maisel et al., 2002; Richards and Troughton, 2004; Reginauld et al., 2019). The elevation of ANP and BNP concentrations in CHF seem to be serve as a beneficial compensatory responsiveness that offset cardiac events (Stevens et al., 1995; Wang et al., 2014). However, the emerging paradigm in hypertension and CVDs is the existence of ANP and BNP deficiency with high BP and CVDs. From the point of view of the roles of ANP, BNP, and their cognate receptor NPRA in hypertension and CVDs, this section has been classified in the follow categories:

(a) Hypertension and Cardiovascular Diseases

(b) Renovascular Dysfunction

(c) Cardiac remodeling and dysfunction

(d) Metabolic Syndrome and Diabetic Conditions

(e) Immunogenic Responses and Cardiovascular Disorders

(a) Hypertension and Cardiovascular Diseases

The activation of ANP-BNP/NPRA signaling lowers fluid volume and BP by causing excretion of salt and water from the kidneys and inhibition of the vasoconstriction of vascular smooth muscle cells. The mutations in genes encoding ANP, BNP, and NPRA seems to exert deleterious effects and trigger high BP and CVDs (Ellmers et al., 2007; Pandey, 2008, 2018; Kishimoto et al., 2011). Several studies have demonstrated a link of ANP (*NPPA*), BNP (*NPPB*), and NPRA (*NPR1*) polymorphisms with hypertension and CVDs in humans (Rubattu et al., 2006; Webber and Marder, 2008; Xue et al., 2008; Newton-Cheh et al., 2009; Vandenwijngaert et al., 2019). In mice, the gene-knockout phenotypes of *Nppa*, *Nppb*, *Nppc*, *Npr1*, *Npr2*, and *Npr3* are provided in Table 1.

**TABLE 1:** Tissue distribution of natriuretic peptides (ANP, BNP, and CNP) along with their cognate receptors (NPRA, NPRB, and NPRC) and gene-knockout phenotypes in mice.

Gene	Protein	Tissue-distribution	Gene-knockout phenotype	References
*Nppa*	ANP	Atrium, brain, kidney, ovary, testis, ventricle	Hypertension, cardiac hypertrophy	Steinhelper et al., 1990; John et al., 1995; Lin et al., 1995; Melo et al., 1999
*Nppb*	BNP	Atrium, brain, ventricle	Ventricular fibrosis, skeletal abnormalities, vascular complications	Ogawa et al., 1994; Tamura et al., 2000
*Nppc*	CNP	Aorta, brain, heart, testis, vascular endothelium	Inhibition of long bone growth, dwarfism, abnormal chondrocyte growth	Chusho et al., 2001; Yasoda et al., 2004; Wang Y. et al., 2007; Moyes et al., 2020
*Npr1*	NPRA	Adrenal glands, brain, heart, kidney, liver, lung, olfactory neurons, ovary, pituitary gland, placenta, testis, thymus, vasculature, liver	Hypertension, cardiac hypertrophy and fibrosis, inflammation, volume overload, reduced testosterone	Lopez et al., 1995; Oliver et al., 1997; Shi et al., 2003; Vellaichamy et al., 2005a; Reinhart et al., 2006; Ellmers et al., 2007; Zhao et al., 2007; Das et al., 2010; Vellaichamy et al., 2014; Subramanian et al., 2016; Das et al., 2020
*Npr2*	NPRB	Brain, cartilage, heart, lung, ovary, pituitary gland, placenta, testis, thymus, vasculature	Dwarfism, decreased adiposity, female sterility, seizures, vascular complications	Tamura et al., 2004; Langenickel et al., 2006
*Npr3*	NPRC	Brain, heart, intestine, kidney, liver, vasculature	Bone deformation, skeletal over-growth, long bone overgrowth	Matsukawa et al., 1999

*ANP, atrial natriuretic peptide; Nppa, coding for pro-atrial natriuretic peptide; BNP, brain natriuretic peptide; Nppb, coding for pro-brain natriuretic peptide; CNP, C-type natriuretic peptide; Nppc, coding for pro-C-type natriuretic peptide. NPRA, natriuretic peptide receptor-A; Npr1, coding for NPRA; NPRB, natriuretic peptide receptor-B; Npr2, coding for NPRB; NPRC, natriuretic peptide receptor-C; Npr3, coding for NPRC.*

Genetically modified mouse models have helped to delineate the functions of ANP, BNP, and NPRA in the physiological and pathophysiological conditions of high BP and CVDs (John et al., 1995; Oliver et al., 1997; Shi et al., 2003; Vellaichamy et al., 2005a; Ellmers et al., 2007; Pandey, 2019). Transgenic mice overexpressing ANP developed sustained hypotension; also their mean arterial pressure (MAP) was decreased by 25−30 mmHg compared with the non-transgenic control groups (Steinhelper et al., 1990; Melo et al., 1999). Somatic delivery of the proANP cDNA to a spontaneously hypertensive rat (SHR) model induced a sustained reduction in systolic BP (Lin et al., 1995). Similarly, overexpression of proANP in hypertensive mice lowered systolic BP, suggesting that the proANP is an ideal gene-therapy candidate for the treatment of human hypertension and CVDs (Schillinger et al., 2005).

Several cardiovascular pathways, including NPs, RAAS, and the adrenergic systems, are considered to regulate BP and cardiovascular events. However the genetic determinants contributing to inter-individual differences in BP regulation have been linked with only the NPs and their receptors (Newton-Cheh et al., 2009). Previously, it was indicated that the polymorphism in the *NPPA* caused left ventricular cardiac hypertrophy in the Italian patients suffering from high BP and these patients also showed significantly reduced levels of proANP (Rubattu et al., 2006). The relationship between BP and cardiovascular disorders indicated that in the absence of ANP-BNP/NPRA signaling, even small increases in BP had excessive and detrimental effects. Mutation of a single allele in the human *NPR1* was found to decrease levels of NPRA protein by 70% and showed susceptibility to BP, kidney dysfunction, and LVH (Nakayama et al., 2000). The single allele mutation in *NPR1* in human could reflect parallel characteristics in the haplotype genotype of *Npr1*^/^ in mice (Shi et al., 2003; Vellaichamy et al., 2005a; Kumar et al., 2017). It has been reported that a positive association exists between the polymorphisms of *NPPA, NPPB, and NPR1* causing essential hypertension and LHV (Newton-Cheh et al., 2009; Vandenwijngaert et al., 2019). Those previous studies indicated that genetic polymorphisms in *NPPA, NPPB, and NPR1* seem to be linked with a family history of high BP, LVH, cardiac mass index, and paraventricular septal wall thickness. More studies will be required to characterize the functionally significant markers of *NPPA, NPPB*, and *NPR1* polymorphism variants in a larger human population.

Global ablation of *Nppa* in mice can increase BP and cause hypertension (John et al., 1995). The genetic mouse model with ablation of *Nppa*, has suggested that ANP has a central role in hypertension. In *Nppa* null mutant (*Nppa^/^)* mice fed standard or intermediate-salt diets, BP was elevated by 8−10 mmHg. Haplotype (*Nppa^/^)* mice on a standard-salt diet contained a normal amount of plasma ANP and had normal BP, while on a high-salt diet these animals were hypertensive, and their BP was elevated by 25−27 mmHg. Global deletion of the *Nppa* allele can lead to salt-sensitive hypertension even when plasma ANP level are not significantly decreased. Global ablation of *Npr1* increases BP by 36−40 mmHg in null mutant (*Npr1*^/^; 0-copy) mice as compared with wild-type (*Npr1^/^;* 2-copy) mice (Oliver et al., 1997; Shi et al., 2001, 2003; Das et al., 2010, 2020; Vellaichamy et al., 2014). In contrast, increased expression of global *Npr1* in gene-duplicated (*Npr1*^/^; 3-copy and *Npr1*^/^; 4-copy) mice significantly reduced BP and enhanced kidney and heart function (Oliver et al., 1998; Shi et al., 2003; Zhao et al., 2013; Das et al., 2020).

Earlier, we examined the mechanisms that may mediate the function of increasing numbers of *Npr1* gene copies, determining the excretion of urine and sodium, renal blood flow (RBF), glomerular filtration rate (GFR), and BP after blood volume expansion in *Npr1* 0-copy, 2-copy, and 4-copy mice in a gene-dose-dependent manner (Shi et al., 2003). The volume expansion with whole blood infusion, increased MAP in *Npr1* gene-knockout (0-copy), wild-type (2-copy), and gene-duplicated (4-copy) mice. In addition, *Npr1* null mutant (0-copy) mice retained significantly higher levels of Na^+^ and water; however, gene-duplicated (4-copy) mice as compared to wild-type (2-copy) mice had greatly reduced levels of Na^+^ and water. Our findings demonstrated that the ANP/NPRA axis is predominantly responsible for regulating the renal hemodynamics and Na^+^ excretory responses to intravascular blood volume expansion.

(b) Renovascular Dysfunction

ANP-BNP/NPRA system primarily affects glomerulus, tubular, and vascular functions in the kidneys (Nonguchi et al., 1987; Kremer et al., 1988; Light et al., 1989; Appel, 1990; Cermak et al., 1996; Kumar et al., 1997b, 2014; Pandey et al., 2000; Tripathi and Pandey, 2012; Das et al., 2020). ANP prevents contraction of mesangial cell and vascular smooth muscle cells in response to vasoconstrictors, including Ang II, endothelin, vasopressin, and agonists of the adrenergic systems (Appel, 1990; Kumar et al., 1997a; Levin et al., 1998; Pandey, 2018). NPRA antagonists, A71915 and HS-121-1 abolished the renal effect of infused ANP, including a decrease in urinary cGMP (von Geldern et al., 1990; Sano et al., 1992). In the kidneys, a combination of hemodynamic and tubular transport effects seem to be responsible for ANP-BNP/NPRA-induced natriuresis and diuresis, exerting both direct and indirect effects on tubules, including the proximal tubules, as well as cortical and innermedullary collecting ducts (Sonnenberg et al., 1986; Brenner et al., 1990; Zhao et al., 2010; Prieto et al., 2012). ANP actions facilitate the excretion of salt and water with an increase in GFR and RBF in the kidneys (Burnett et al., 1984; Camarago et al., 1984; Freeman et al., 1985; Meyer and Forsmann, 1997; Villrreal and Freeman, 1997; Melo et al., 2000; Shi et al., 2003; Pandey, 2008; Zhao et al., 2010). ANP- and BNP-induced natriuresis and diuresis causes a direct inhibition of tubular transport processes. This occurs without dependence on alterations in GFR and RBF, which favor both hemodynamic and tubular effects (Sonnenberg et al., 1986; Meyer et al., 1997). The natriuretic and diuretic actions of ANP and BNP lead to the direct inhibition of tubular transport mechanisms. It has been proposed that ANP-BNP/NPRA system also interacts with another local natriuretic system, by enhancing the action of renal dopaminergic system (Choi et al., 2014). These authors suggested that part of the inhibitory effects of ANP on sodium and water reabsorption depends on dopaminergic system. Those previous studies also indicated that ANP/NPRA signaling stimulates dopamine uptake in tubular cells and also reduces the dopamine catabolism. Similarly, urodialatin also seem to exert similar effect on renal dopaminergic system (Citarella et al., 2009; Choi et al., 2013). Confocal immunofluorescence microscopic studies have indicated that the epithelial sodium channel (ENaC) is regulated by ANP/NPRA-dependent second messenger cGMP in *Xenopus* 2F3 cells (Guo et al., 2013). The authors of those previous studies suggested that NPRA, but not NPRB or NPRC, is involved in the regulation of ENaC activity. Further, with the increasing concentrations of intracellular cGMP, ENaC activity was inhibited in epithelial cells from patients with cystic fibrosis (Poschet et al., 2007).

The ANP-BNP/NPRA signaling cascade plays critical roles to counteract both systemic and local RAAS (Meyer and Forsmann, 1997; Shi et al., 2001; Pandey, 2005a, 2011; Vellaichamy et al., 2007). ANP/NPRA markedly lowers renin secretion from the kidneys and also reduces plasma renin concentrations (Burnett et al., 1984; Obana et al., 1985; Paul et al., 1988; Meyer and Forsmann, 1997; Shi et al., 2003). Further, using genetically modified mouse models, ANP/NPRA signaling has been shown to suppress renin activity and other RAAS components as well as to decrease BP (Obana et al., 1985; Brenner et al., 1990; Levin et al., 1998; Shi et al., 2001; Pandey, 2005a; Vellaichamy et al., 2007; Periyasamy et al., 2019).

Studies from our laboratory using *Npr1*^–/–^ null mutant mice have demonstrated that at the birth, the absence of NPRA allows increased renin mRNA expression and greater renin and Ang II levels than occur in *Npr1*^–/–^ wild-type mice (Shi et al., 2001). On the contrary, in adult *Npr1*^–/–^ mice, both circulating and intrarenal concentrations of renin and Ang II levels were significantly lower than those in wild-type control animals. The decrease in renin concentrations in adult *Npr1*^–/–^ mice was found to be due to progressive increases in BP, which lead to inhibition of renin synthesis and secretion from juxtaglomerular cells in the kidney. It has been suggested that an increased levels of ANP released into the circulation in response to blood volume expansion, was mainly responsible for the natriuretic and diuretic actions (Paul et al., 1988; Antunes-Rodrigues et al., 1992). We have examined the quantitative contributions and possible mechanisms mediating renin synthesis and release using the varying *Npr1* gene copy of *Npr1*^–/–^ (0-copy), *Npr1*^+/+^ (2-copy), and *Npr1*^++/++^ (4-copy) mice in a gene-dose-dependent manner (Shi et al., 2003). Our studies have demonstrated that NPRA ablation increases the expression of AT_1_R and angiotensin-converting enzyme 1 (ACE 1) in the kidneys of *Npr1*^–/–^ null mutant mice (Periyasamy et al., 2019).

ANP-BNP/NPRA signaling inhibits the synthesis and secretion of aldosterone in adrenal glomerulosa cells suggesting that ANP is important for natriuretic and diuretic responses (Atarashi et al., 1984; Brenner et al., 1990; Anand-Srivastava and Trachte, 1993; Pandey, 2005a). The NPRA antagonist HS-142-1 was found to eliminated the suppressive effects of ANP on aldosterone synthesis and secretion in adrenal glomerulosa cells (Oda et al., 1992). The ANP/NPRA system increases cGMP concentrations, resulting in an activation of cAMP-dependent phosphodiesterases, which decreases both cAMP and aldosterone levels in adrenal glomerulosa cells (MacFarland et al., 1991; Levin et al., 1998; Pandey, 2005a).

Interestingly, adrenal renin content and renin mRNA along with Ang II and aldosterone levels were found to be increased in the adult *Npr1*^–/–^ mice than with *Npr1*^+/+^ control animals (Shi et al., 2001; Zhao et al., 2007). ANP-BNP/NPRA signaling opposes all the actions of Ang II in the phathophysioogical states (Pandey, 2005a, 2010). Our studies demonstrated that ablation of *Npr1* exhibits in chronic elevation of BP in mice fed a high-salt diet (Oliver et al., 1998; Zhao et al., 2007, 2013). On the other hand, adrenal Ang II and aldosterone concentrations were decreased in *Npr1* gene-duplicated animals kept on the high-salt diet; however, a low-salt diet increased the adrenal Ang II and aldosterone concentrations in *Npr1* mice in a gene-dose-dependent manner (Zhao et al., 2007, 2013). Previously, it was indicated that BP of *Npr1*^–/–^ mice remained at higher levels and unchanged in response to high-salt diets (Lopez et al., 1995). Our findings indicated that NPRA signaling is protective against the effects of a high-salt diet in the kidneys and heart of *Npr1* gene-duplicated mice (Zhao et al., 2007, 2013).

(c) Cardiac Remodeling and Dysfunction

Plasma ANP and BNP levels are markedly elevated in the pathophysiological and clinical conditions of cardiac dysfunction and remodeling, including fibrosis, diastolic dysfunction, cardiac hypertrophy, pulmonary embolism, and CHF leading to severe conditions of CVDs (Vellaichamy et al., 2005a, 2007; Felker et al., 2006; Jaffe et al., 2006; Reinhart et al., 2006; See and de Lemos, 2006; Ellmers et al., 2007; Zhao et al., 2013; Rubattu and Volpe, 2014; Pandey, 2019). ANP and BNP exert their cardioprotective functions not only as the circulating peptide hormones but also as local autocrine and paracrine factors (Chen et al., 2019; Pandey, 2019). ANP-BNP/NPRA signaling serves cardiac protective role by various mechanisms (Table 2). The concentrations of ANP and BNP are increased proportion to the severity of cardiac dysfunction and remolding in humans and experimental animal models (Nakao et al., 1996; Chen and Burnett, 1999; Reinhart et al., 2006; Ellmers et al., 2007; Pandey, 2008; Rubattu et al., 2019). The concentrations of ANP and BNP are also markedly elevated in the cardiac tissues of patients with CHF. In humans, both of these hormones appear to reduce the preload and afterload of the heart. The high plasma levels of ANP and BNP tend to predict cardiac events and CHF but BNP levels rise 10−25-fold higher than the fold increases in ANP concentrations (Mukoyama et al., 1991; Tsutamoto et al., 1993; Chen and Burnett, 1999; Felker et al., 2006; Reinhart et al., 2006; Pandey, 2019; Volpe et al., 2019a). *NPPA*, *NPPB*, and *NPR1* genes are overexpressed in hypertrophied failing heart, suggesting that the autocrine and/or paracrine effects of NPs predominate and acts endogenously to protect against maladaptive pathology of CVDs (Knowles et al., 2001; Vellaichamy et al., 2005a, 2007, 2014; Felker et al., 2006; Ellmers et al., 2007; Xue et al., 2008; Subramanian et al., 2016; Pandey, 2019; Volpe et al., 2019a, b).

**TABLE 2:** Effect of ANP-BNP/NPRA signaling in different cardiovascular target organs and tissues.

Tissues	Parameters	References
Heart	Increased fractional shortening Decreased heart weight/body ratio Decreased LVDS and LVDD Inhibition of cardiac hypertrophy Inhibition of fibrosis Inhibition of inflammation	Oliver et al., 1997; Ellmers et al., 2002; Vellaichamy et al., 2005a; Zhao et al., 2013; Vellaichamy et al., 2014; Subramanian et al., 2016
Kidney	Increased GFR and RBF Increased natriuresis/diuresis Decreased Na and water transport Decreased Na absorption Decreased solute concentration Inhibition of renin release Inhibition of hypertrophy Inhibition of remodeling Inhibition of fibrosis Inhibition of inflammation	de Bold et al., 1981; Burnett et al., 1984; Freeman et al., 1985; Obana et al., 1985; Sonnenberg et al., 1986; Light et al., 1989; Appel, 1990; Meyer and Forsmann, 1997; Shi et al., 2001; Shi et al., 2003; Das et al., 2010; Kumar et al., 2017; Gogulamudi et al., 2019; Das et al., 2020
Vasculature	Increased smooth muscle relaxation Increased endothelial permeability Decreased intravascular volume Inhibition of cell growth	Garcia et al., 1984; Abell et al., 1989; Itoh et al., 1990; Appel, 1990, 1992; Sharma et al., 2002; Sabrane et al., 2005; Sen et al., 2016; Arise et al., 2020
Adrenal gland	Inhibition of aldosterone release Inhibition of renin synthesis	Shi et al., 2001; Airhart et al., 2003

*ANP, atrial natriuretic peptide; BNP, brain natriuretic peptide; NPRA, natriuretic peptide receptor-A; LVDS, left ventricular dimension systolic; LVDD, left ventricular dimension diastolic; RBF, renal blood flow; GFR, glomerular filtration rate.*

The longer half-life of BNP has favored its diagnostic use over that of ANP to evaluate NPs as important indicator of CHF in patents with chest pain in emergency conditions (Reinhart et al., 2006; Rubattu and Volpe, 2014). The ventricular expression of *Nppa*, *Nppb*, and *Npr1* is more closely associated with local cardiac hypertrophic and fibrotic events than are plasma ANP levels and systemic BP (Vellaichamy et al., 2005a, 2007). Nevertheless, NT-pro-BNP seems to be a stronger predictor of cardiovascular risk (Doust et al., 2005; Khan et al., 2006; Girsen et al., 2007; Kocylowski et al., 2009). In hypertrophied and failing hearts, the expression of *Nppa* and *Nppb*, which serve as endogenous protective mechanisms against maladaptive cardiac events and dysfunction is markedly increased (Knowles et al., 2001; Zahabi et al., 2003; Vellaichamy et al., 2005a; Pandey, 2019; Rubattu et al., 2019). The alterations in *Nppa* promoter seem to be linked with cardiac hypertrophy and dysfunction (Deschepper et al., 2001). Mice lacking NPRA develop cardiac hypertrophy and fibrosis, independent of BP (Oliver et al., 1997; Nakanishi et al., 2005; Vellaichamy et al., 2005a, 2007, 2014; Ellmers et al., 2007; Scott et al., 2009; Zhao et al., 2013). Our studies have demonstrated that the global ablation of *Npr1* in mice triggers the expression of hypertrophic and fibrotic markers and matrix metalloproteinases (MMP-2, MMP-9) in the cardiac tissues (Vellaichamy et al., 2005a, 2007, 2014; Pandey, 2008, 2011; Subramanian et al., 2016). It has been indicated that ANP/NPRA signaling antagonizes Ang II-induced collagen systhesis via suppression and activation of MMP-2, MMP-9 (Parthasarathy et al., 2013).

**FIGURE 2:**
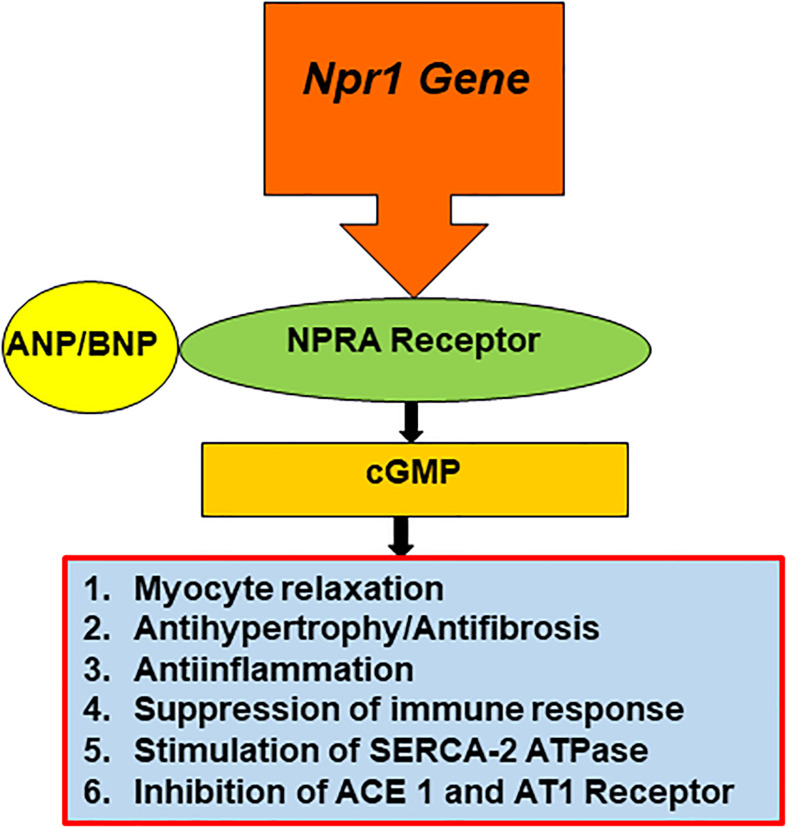
The schematic representation of the NP-dependent activation and signaling of ANP/NPRA in the heart. To maintain the cardiac homeostasis, *Npr1* gene is transcribed and translated to produce mature NPRA protein. Specifically, NPRA is activated by ANP and BNP; in turn produces intracellular second messenger cGMP that activates various physiological responsiveness in the heart tissues and cells.

The expression of Ca^2+^-ATPase-2a (SERCA-2a) is progressively decreased and the level of cytosolic Ca2^+^ is increased in the hypertrophied heart of *Npr1*^–/–^ mice (Vellaichamy et al., 2005a; Pandey and Vellaichamy, 2010). ANP-BNP/NPRA signaling mechanism is known to decrease the cytosolic levels of Ca2^+^ and inosital trisphosphate in different cells and tissues (Pandey, 2014). Further, both ACE 1 and AT_1_R were found to be significantly increased leading to cardiac hypertrophy and fibrosis in *Npr1*^–/–^ mice, but not *Npr1*^+/+^ control mice (Vellaichamy et al., 2007). In failing hypertrophied hearts, ANP-BNP/NPRA signaling antagonizes Ang II- and AT_1_R receptor-mediated cardiac dysfuction and remodeling (Li et al., 2002; Kilic et al., 2007; Vellaichamy et al., 2007; Zhao et al., 2013). The endothelial cell-specific arteries from *Npr1* gene-disrupted mice showed significant elevaion in systolic BP (Sabrane et al., 2005). The conditional inactivation of *Npr1* in cardiac myocytes, exhibited mild cardiac hypertrophy; however, ANP levels were markedly increased (Holtwick et al., 2003). Both ANP and BNP levels were elevated in the global *Npr1^–/–^* and haplotype *Npr1*^+/–^ mice with myocardial infarction. These mice showed a higher incidence of CHF and significantly greater mortality rates than did wild-type mice (Nakanishi et al., 2005; Vellaichamy et al., 2005a, 2007). ANP-BNP/NPRA signaling provides cardiac protective mechanism against maladaptive cardiac disorders and remodeling of CVDs (Figure 2).

(d) Metabolic Syndrome and Diabetic Conditions

The growing evidence suggest that ANP-BNP/NPRA signaling regulates whole body metabolism and controls diabetic conditions (Wang et al., 2004; Wang T.J. et al., 2007; Moro, 2013; Coue et al., 2015). The previous studies have indicated that a decreased plasma levels of ANP might be associated with the obesity, metabolic syndrome, insulin resistance, energy balance, and glucose homeostasis in humans (Wang et al., 2004; Wang T.J. et al., 2007; Birkenfeld et al., 2008; Coue and Moro, 2016; Cannone et al., 2019). It has also been shown that NPs signaling activates the peroxisome proliferator-activated receptor-γ (PPAR-γ) and enhances the fat oxidation and mitochondrial biogenesis in the muscle tissues (Mitsuishi et al., 2008; Miyashita et al., 2009). The ANP/NPRA signaling also enhances the PPAR-γ coactivator-1α (PGC-1α), which stimulates oxidative phosphorylation (OXPHOS) in skeletal muscle tissues (Engeli et al., 2012). The previous studies have demonstrated that ANP/NPRA system accelerates lipid mobilization, mitochondrial oxidative metabolism, and fat oxidation (Birkenfeld et al., 2008, 2012; Tsukamoto et al., 2009). In fact, ANP/NPRA action modulates energy expenditure and supply of fatty acids for cardiac and skeletal muscle metabolic utilization (Engeli et al., 2012; Schlueter et al., 2014).

Furthermore, ANP/NPRA signaling seems to regulate obesity, type 2 diabetes, and resistance to insulin (Moro, 2013; Coue et al., 2015). On the other hand, previous studies have indicated that NPRC exhibits lipolytic effect of NPs in mouse adipose tissues (Sengenes et al., 2002; Bordicchia et al., 2012). Mice challenged to the low temperature environment showed stimulated release of ANP, but exhibited a reduced level of NPRC in both brown and white adipocytes (Bordicchia et al., 2012). Interestingly, ANP has been shown to enhance the browning of human white adipose tissues (Miyashita et al., 2009; Enerback, 2010). It has been suggested that insulin enhances the expression of NPRC in adipocytes in a glucose-dependent manner (Bordicchia et al., 2016). It is believed that a defective ANP-BNP/NPRA signaling might promote a maladaptive metabolic disorders, which could trigger lipid accumulation, decreased mitochondrial function, hyperglycemia, and insulin resistance leading to hypertension and CVDs in humans. It is believed that an increased circulating concentrations of ANP might serve as protective mechanisms, nevertheless, the human subjects having low circulating levels of ANP might have a greater risk of cardiometabolic syndrome (Pereira et al., 2015).

(e) Immunogenic Responses and Cardiovascular Disorders

Proinflammatory cytokines play a central role in the development of hypertension, cardiac hypertrophy, and CHF in experimental animal models and in humans (Hirota et al., 1995; Testa et al., 1996; Vanderheyden et al., 2005; Vellaichamy et al., 2005a, 2014; Das et al., 2010, 2020; Subramanian et al., 2016; Gogulamudi et al., 2019). Elevated circulating and myocardial levels of proinflammatory cytokines, including tumor necrosis factor (TNF-α), interleukin-1β (IL-1β), and interleukin-6 (IL-6) have been reported in the plasma of cardiomyopathic patients, correlating with the severity of their disease (Testa et al., 1996; Vanderheyden et al., 2005). Myocardial cells are capable of producing substantial amounts of proinflammatory cytokines in response to ischemia and experimental load-induced stress (Baumgarten et al., 2002; Palmieri et al., 2002). Inappropriate activation of TNF-α, IL-1β, and IL-6 has been shown to induce phenotypic changes in CVDs, encompassing the myocyte hypertrophy, myocardial apoptosis, extracellular matrix deposition, and contractile dysfunction (Thaik et al., 1995; Sekiguchi et al., 2004; Vellaichamy et al., 2005a, 2014; Subramanian et al., 2016).

Investigations in our laboratory have indicated that the ANP-BNP/NPRA system acts as a negative regulator of inflammation and hypertrophic growth in the kidneys and heart (Vellaichamy et al., 2005a, b, 2007, 2014; Das et al., 2010, 2020; Subramanian et al., 2016; Kumar et al., 2017). ANP inhibited TNF-α production in interferon-gamma (IFN-γ)-activated macrophages and blocked TNF-α-induced adhesion molecule expression in endothelial cells (Tsukagoshi et al., 2001; Vollmer, 2005). We have used *Npr1* gene-knockout and gene-duplicated mouse models in efforts to determine whether genetically determined differences in *Npr1* expression changes the levels of cardiac proinflammatory cytokines (Zhao et al., 2013; Vellaichamy et al., 2014; Subramanian et al., 2016). Our findings suggested that ablation of *Npr1* triggered a sustained activation of proinflammatory cytokines gene expression and protein levels associated with exaggerated ventricular remodeling and cardiac hypertrophy leading to CVDs. However, gene-duplication of *Npr1* in mice attenuated cardiac proinflammatory cytokines expression and protected against cardiac remodeling (Vellaichamy et al., 2005a, 2007, 2014; Subramanian et al., 2016).

Increased expression of TNF-α, IL-6, TGF-β, LT-β, and IFN-γ mRNA and protein levels were found in the hearts of adult *Npr1*^–/–^ mice as compared with the hearts of adult *Npr1*^+/+^ mice (Vellaichamy et al., 2014). In parallel, cytokine receptor protein levels were also increased in the heart of null mutant mice indicating increased expression of gb-130, TNF-α receptor 1 (TNF-αR1), and TNF-β receptor 1 (TGF-βR1). In contrast, significant decreases in IL-6, TNF-α, and TGF-β1 cytokine protein levels were found in the gene-duplicated hearts of *Npr1*^++/++^ mice as compared with the hearts of wild-type mice.

The activated ANP-BNP/NPRA system may serve a protective function by inhibiting the ventricular expression of proinflammatory cytokines in CVDs (Vellaichamy et al., 2005a, b, 2014). Some previous observations advanced the notion that disruption of the ANP/NPRA/cGMP signaling pathway can augment activation of proinflammatory cytokines leading to extracellular matrix remodeling, pathological hypertrophy, and CHF. Expression of myocardial proinflammatory cytokine genes, including TNF-α, IL-1β, and IL-6 was found to be significantly higher in patients with compensated CHF condition, along with increased cytokine gene expression, which could play an adaptive role in left ventricular remodeling and other CVDs (Oral et al., 2003; Vanderheyden et al., 2005). It has been shown that cardiac-specific over expression of proinflammatory cytokines in mouse hearts leads to cardiac hypertrophy and ventricular disorders similar to those in human heart disease, indicating that proinflammatory cytokines are critically involved in the cardiac remodeling process in CVDs (Hirota et al., 1995; Krown et al., 1996; Oral et al., 2003; Vellaichamy et al., 2005a, 2014; Subramanian et al., 2016). Blockade of the action of proinflammatory cytokines has been shown to prevent hypertension, cardiac hypertrophy, and diastolic dysfunction in experimental animal models (Villarreal and Dillmann, 1992; Krown et al., 1996; Isono et al., 1998; Kurrelmeyer et al., 2000; Li et al., 2000; Vellaichamy et al., 2005a, 2007).

**FIGURE 3:**
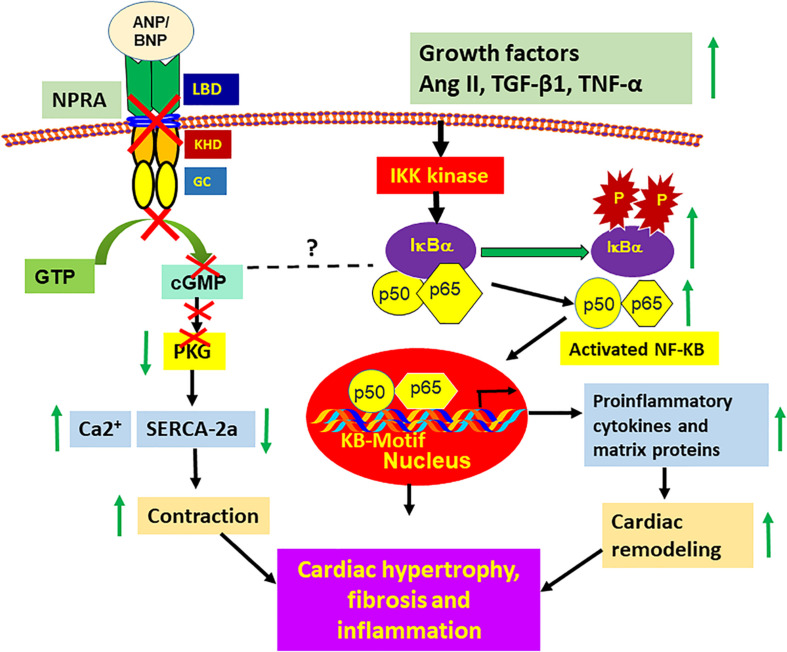
Ablation of *Npr1* triggers the activation of proinflammatory pathways that promotes cardiac hypertrophy, fibrosis, and remodeling. Disruption of ANP/NPRA signaling causes unbalanced activation of master transcription factor NF-κB that initiates the expression of proinflammatory cytokines and matrix proteins; thereby promotes specific cardiac structural and molecular changes in *Npr1*^–/–^ mice heart. The activated NF-κB translocates into the nucleus and promotes the genes transcription and expression of various proinflammatory cytokines and extracellular matrix proteins, including MMP-2 and MMP-9. Increased activation of NF-κB, thereby promotes medial thickening and perivascular fibrosis, in turn leads to the cardiac hypertrophy, fibrosis, remodeling and heart failure. On the other hand, the absence of ANP/NPRA signaling also simultaneously inhibits SERCA-2a that increases the cytosolic Ca2^+^ levels, thereby results in the increased contractile activity of the heart, which also promotes cardiac hypertrophy and remodeling. Ang II, angiotensin II; TGF-β1, transforming growth factor-β1; TNF-α, tumor necrosis factor-α; IKK, inhibitory kappa kinase; IKB, inhibitory kappa B, NF-κB, nuclear factor-kappa B; PKG, cGMP-dependent protein kinase; SERCA-2a, sarcolemmal endoplasmic reticulum Ca2^+^-ATPase-2a; MMP-2 and -9, matrix metalloproteinase-2 and −9.

Evidence suggests that the ANP-BNP/NPRA system has antiinflammatory activity, and could inhibit the bacterial toxin (LPS)- and IFN- gamma- induced expression of proinflammatory cytokines and nuclear factor-kappa B (NF-κB) in macrophages (Tsukagoshi et al., 2001; Vollmer, 2005). Ablation of *Npr1* also enhances the expression and activation of transcription factors, NF-κB and activating protein-1 (AP-1), which seem to be associated with cardiac hypertrophy, fibrosis, and extracellular matrix remodeling (Vellaichamy et al., 2005a, 2007, 2014). *Npr1* gene-disruption activates NF-κB and leading to cardiac remodeling and hypertrophic conditions (Figure 3). A significant increases in mRNA expression and protein levels of NF-κB and IKK-β isoforms were observed in the hearts and kidneys of *Npr1*^–/–^ and *Npr1*^+/–^ mice as compared with *Npr1*^+/+^mice, suggesting that the NF-κB signaling pathway is activated in the hearts and kidneys of mutant mice (Vellaichamy et al., 2005a, 2014; Subramanian et al., 2016). The increased NF-κB binding activity was positively correlated with increased expression of proinflammatory cytokine genes in *Npr1* null mutant mice with initiation and development of CVDs (Vellaichamy et al., 2005a, 2014; Subramanian et al., 2016).

Previous studies indicated that the activation of NF-κB and proinflammatory cytokines serve as a causal event in the development of cardiac hypertrophy and fibrosis (Frantz et al., 2003; Purcell and Molkentin, 2003; Li et al., 2004; Vellaichamy et al., 2005a; Subramanian et al., 2016). The sustained activation of proinflammatory cytokines contributes to the pathological forms of ventricular remodeling and exaggerating the CVDs in experimental animals (Leitman et al., 1988; Hirota et al., 1995; Baumgarten et al., 2002; Palmieri et al., 2002; Vellaichamy et al., 2005a, 2014; Zhao et al., 2013). Our findings suggested that enhanced ANP-BNP/NPRA signaling can protect the heart by inhibiting ventricular expression of NF-κB, a master regulator of proinflammatory cytokines in relation to increasing *Npr1* gene copies (Vellaichamy et al., 2014; Subramanian et al., 2016).

## Perspectives

In the past four decades, a large body of research has provided a unique perspective on the biochemical, cellular, molecular, genetic, and clinical aspects of NPs and their receptors in relation to CVDs. The physiological and pathophysiological roles of ANP-BNP/NPRA signaling are implicated with protective mechanisms in various organ systems, including the heart, kidneys, lungs, central nervous system, gonads, adrenal glands, and vasculature. Cardiac hormones, ANP and BNP are considered to be diagnostic markers of CHF, but we need to determine their therapeutic potentials for the treatment and prevention of CVDs such as hypertension, renal insufficiency, cardiac hypertrophy, CHF, and stroke. Intraperitoneal delivery of recombinant ANP (Carperitide) facilitated the recovery of increased blood flow in ischemic conditions and exerted antihypertrophic and antifibrotic effects (Suwa et al., 2005; Park et al., 2008; Nishikimi et al., 2013). Recombinant BNP (Nesiritide) exhibited natriuretic and vasodilatory action in CHF patients (Colucci et al., 2000). In *Npr1*^–/–^ gene ablated mice, impaired recovery of blood flow after hand-limb ischemia was significantly inhibited by both ANP and BNP (Tokudome et al., 2009). Genetic molecular approaches have delineated of the functions of NPRA by decreasing and/or increasing *Npr1* gene-copies (gene-knockout and/or gene-duplication) in mice by genetically altering protein product levels. It has been found that common genetic variants of *NPPA, NPPB*, and *NPR1* are associated with circulating ANP and BNP levels and BP, contributing individual variations in the regulation of BP and CVD risk factors (Wang, 2018; Reginauld et al., 2019).

The strategy of enhancing endogenous NPs and their protective roles in CHF is achieved with the drug sacubitril/valsartan, which augments circulating ANP and BNP by inhibiting of neprilysin that degrades NPs (McMurray et al., 2014). This drug combines dual properties; inhibiting both neprilysin and Ang II type 1 receptor (AT_1_R) blocker, which is also referred to as Ang II receptor-neprilysin inhibitor (ARNi). Enhancing endogenous ANP-BNP/NPRA signaling has proven to be critical in the first line of therapeutic targets for hypertension, cardiac dysfunction, and CHF in decades (McMurray et al., 2014).

The apparent action of ANP-BNP/NPRA system in antagonizing the RAAS, there seem to be immense potential in using the NPs as a novel therapeutic axis in treating hypertension, renal inefficiencies, and CVDs. The clinical trials have suggested both the benefits and risks of using the synthetic ANP (anaritide and carperitide) and BNP (nesiritide) for the treatment of hypertension, renal diseases and CVDs. Unfortunately, synthetic NPs as a drug have not yet been recommended for the therapeutic treatments in the United States (Kuhn et al., 2009; Saito, 2010; Hayek and Nemer, 2011; Dohi and Ito, 2014; McMurray et al., 2014). However, the success of the drug sacubitril/valsartan in treating the CHF has heightened the enthusiasm and interest in NPs as a therapeutic target for hypertension, renal diseases, and CVDs. This drug combines an inhibitor of the NPs degrading enzyme neprilysin (sacubitril) with an antagonist of Ang II receptor 1 (AT_1_R) (Valsartan), which lower BP and pulse pressure (Vellaichamy et al., 2005a; Ruilope et al., 2010; Williams et al., 2017). Interestingly, the new chimeric natriuretic peptide-based therapies have a great potential in treating hypertension and CVDs. Furthermore, molecular mimicking of the action of BP-lowering Npr1 expression and receptor signaling could be effective in identifying the novel mechanisms for the new therapeutic treatment strategies.

Future studies are expected to lead a better understanding of the genetic and molecular basis of the ANP-BNP/NPRA system in regulating CVDs, including high BP, stroke, CHF, and neurological disorders. The results of future investigations of NPs and their receptors should certainly help resolve the complexities of CVDs. These future studies need to be designed to elucidate the genetic and molecular basis of *Npr1* function in both normal and disease states. Both ongoing and future clinical studies will be needed to identify more functionally significant markers of *NPPA, NPPB*, and *NPR1* variants in a larger human population. The chimeric designer peptides of ANP, BNP, and DNP have opened new avenues for studies of CHF, cardiac disorders, and remodeling therapy. The future progress in this area of research should significantly strengthen and advance our understanding of the genetic and molecular approaches used to evaluate diverse pathophysiological conditions in CVDs. We need to expand the potential clinical implications of ongoing investigations on the next generation personalized medicine and pharmacogenomics of NPs and their receptors that are currently in progress. The resulting knowledge will yield novel therapeutic targets and new treatment strategies for CVDs.

## Author Contributions

KP wrote the manuscript and complied information and references.

## Conflict of Interest

The author declares that the research was conducted in the absence of any commercial or financial relationships that could be construed as a potential conflict of interest.

## Publisher’s Note

All claims expressed in this article are solely those of the authors and do not necessarily represent those of their affiliated organizations, or those of the publisher, the editors and the reviewers. Any product that may be evaluated in this article, or claim that may be made by its manufacturer, is not guaranteed or endorsed by the publisher.
